# Sustained Delivery of Lactoferrin Using Poloxamer Gels for Local Bone Regeneration in a Rat Calvarial Defect Model

**DOI:** 10.3390/ma15010212

**Published:** 2021-12-28

**Authors:** Young Eun Park, Kaushik Chandramouli, Maureen Watson, Mark Zhu, Karen E. Callon, Donna Tuari, Hani Abdeltawab, Darren Svirskis, David Shaun Musson, Manisha Sharma, Jillian Cornish

**Affiliations:** 1 Bone and Joint Research Group, Department of Medicine, School of Medicine, The University of Auckland, Auckland 1023, New Zealand; y.park@auckland.ac.nz (Y.E.P.); m.watson@auckland.ac.nz (M.W.); mzhu031@aucklanduni.ac.nz (M.Z.); k.callon@auckland.ac.nz (K.E.C.); DTuari@bionicsinstitute.org (D.T.); d.musson@auckland.ac.nz (D.S.M.); 2 School of Pharmacy, The University of Auckland, Auckland 1023, New Zealand; kaushikc_32@yahoo.in (K.C.); h.abdeltawab@auckland.ac.nz (H.A.); d.svirskis@auckland.ac.nz (D.S.); 3 Department of Surgery, Auckland District Health Board, Auckland 1023, New Zealand; 4 Department of Nutrition, School of Medical Sciences, The University of Auckland, Auckland 1023, New Zealand

**Keywords:** lactoferrin, LF, poloxamers, poloxamer 407, poloxamer 188, thermoresponsive gels, bone regeneration, rat calvarial defect, hydrogel

## Abstract

Lactoferrin (LF) is a multifunctional milk glycoprotein that promotes bone regeneration. Local delivery of LF at the bone defect site is a promising approach for enhancement of bone regeneration, but efficient systems for sustained local delivery are still largely missing. The aim of this study was to investigate the potential of the poloxamers for sustained delivery of LF to enhance local bone regeneration. The developed LF/poloxamer formulations were liquid at room temperature (20 °C) transforming to a sustained releasing gel depot at body temperature (37 °C). In vitro release studies demonstrated an initial burst release (~50%), followed by slower release of LF for up to 72 h. Poloxamer, with and without LF, increased osteoblast viability at 72 h (*p* < 0.05) compared to control, and the immune response from THP-1 cells was mild when compared to the suture material. In rat calvarial defects, the LF/poloxamer group had lower bone volume than the controls (*p* = 0.0435). No difference was observed in tissue mineral density and lower bone defect coverage scores (*p* = 0.0267) at 12 weeks after surgery. In conclusion, LF/poloxamer formulations support cell viability and do not induce an unfavourable immune response; however, LF delivery via the current formulation of LF200/poloxamer gel did not demonstrate enhanced bone regeneration and was not compatible with the rat calvarial defect model.

## 1. Introduction

Lactoferrin (LF) is a glycoprotein that is present in milk and exocrine secretions in mammals. It has antimicrobial and immunomodulatory properties and can increase bone growth [1]. Previously, bovine LF was found to increase osteoblast proliferation and differentiation, protect osteoblasts from apoptosis and inhibit osteoclastogenesis [2]. In vivo, local or systemic injection of LF enhanced bone formation in calvaria [2,3]. Moreover, local delivery of LF in a hydrogel or microspheres improved bone regeneration in rodent calvarial defects and pig frontal bone [4,5,6].

LF has the potential to be developed as a therapeutic agent to treat bone defects and fractures. LF is an 80-kDa protein made up of a single polypeptide chain containing about 690 amino acids [7,8,9]. However, it is susceptible to rapid enzymatic degradation in the body with a very high clearance [10,11,12]. Under such circumstances, it is more desirable to deliver LF locally, preferably by developing a drug delivery system that can provide controlled release of LF for a longer time period at the site of administration. For instance, LF is known to have a short half-life; approximately 12 min in rat plasma [13], and a study in rats demonstrated that the subcutaneous administration of LF in solution form provided LF release for up to 5 days, whereas subcutaneous implantation of LF in a hydrogel system provided LF release for up to 14 days [4].

Poloxamers are Food and Drug Administration (FDA)-approved polymers that are widely used as vehicles for controlled drug delivery. Poloxamers are thermoresponsive, water-soluble triblock copolymers comprised of poly(ethylene oxide) (PEO) and poly(propylene oxide) (PPO) units. The outer regions consist of hydrophilic PEO units, and the inner core is made up of hydrophobic PPO units [14]. Poloxamer can be formulated as in situ aqueous gels, which are liquid at room temperature and transform immediately into a gel depot at body temperature following administration. The polarity of PEO-PPO blocks in poloxamer changes with increase in the temperature. Poloxamers are more soluble at cold temperatures as it forms stable hydrogen bonds with water molecules. As the temperature rises, and at critical micelle temperatures, the hydrogen bonds are weakened and poloxamer self-assemble to form spherical micelles. With further increase in the temperature, micelles grow and entangle to form a three-dimensional gel network structure [15,16]. Due to the amphiphilic nature of the poloxamer, they can carry both hydrophobic and hydrophilic drugs.

Poloxamers have found use in a number of clinical applications due to their low toxicity and low immunogenicity properties [14]. Poloxamers, specifically poloxamer 407 (P407) and poloxamer 188 (P188), are used to treat endovascular occlusion of blood vessels and sickle cell disorders/vaso-occlusive crisis, respectively [17,18]. Poloxamers are widely used for targeted drug delivery, including the delivery of chemotherapeutic drugs, analgesics, anaesthetics, antibiotics and other proteins and peptides [16,19,20,21,22,23,24,25]. Therefore, this study aimed to determine the potential of poloxamer based gels as a sustained delivery system for LF to enhance bone regeneration following local administration. Poloxamer gels containing varying concentrations of LF were developed and extensively characterised for its in vitro and in vivo properties.

## 2. Materials and Methods

### 2.1. Materials

Bovine LF was kindly provided by Fonterra (Auckland, New Zealand). Kolliphor^®^ P188 (Poloxamer P188) and Pluronic^®^ F-127 (Poloxamer P407) were obtained from Sigma Aldrich (St. Louis, MO, USA). Water used throughout the study was obtained from the Millipore system (Millipore, Bedford, OH, USA) by reverse osmosis (0.22 μm, Millipore, Bedford, OH, USA).

#### Preparation of the Poloxamer Gels

Poloxamer-based gel formulations were prepared by the cold method, as previously described [24]. Briefly, predetermined weights of P407 (25% *w*/*w*) and P188 (17% *w*/*w*) were dissolved in specified amounts of water at 4 °C by stirring overnight. LF was dissolved at varying concentrations: 10 µg/mL (LF10/poloxamer), 50 µg/mL (LF50/poloxamer), 100 µg/mL (LF100/poloxamer) and 200 µg/mL (LF200/poloxamer). The final formulations were stirred for 2 h, labelled accordingly and kept in the refrigerated walk-in cold room (Model BM 57-XPZ(HFKESC-2-SC), Patton, Auckland, New Zealand) at 4 °C until further use.

### 2.2. Sol-to-Gel Transition Temperature

The gelation temperature was measured using the tube inversion method [26,27]. Briefly, samples (0.5 mL) were transferred to 2-mL Eppendorf tubes equilibrated at 20 °C using a thermomixer. The formulations were then subjected to a temperature ramp from 20–40 °C at 1 °C increments and a hold period of 5 min at each temperature. Additionally, a 5-min equilibration at room temperature was given between every 1 °C increase (a total of 10 min run for a sample at each temperature point). The Eppendorf tubes were taken out and inverted. If the formulation ceased to flow for 10 s, it was said to have gelled, and the temperature was noted as the gelation temperature. Measurements were performed in triplicate for each of the formulations.

#### Gelation Time

The gelation time was also measured using the tube inversion method with a slight modification [27,28]. Samples of the formulations (0.5 mL) were transferred to 2-mL Eppendorf tubes and placed in the Eppendorf Thermomixer C (Hamburg, Germany) equilibrated at a previously determined gelation temperature. The samples were removed at regular time intervals of 30 s, 1, 2, 3, 4 and 5 min and checked for gelation using the tube inversion method. The two time points between which the gelation occurred were selected, and the gelation was reassessed. For the second time, an interval of 10 s was used to determine the specific time point at which the formulation gelled. A digital timer was used to record the gelation time. Measurements were performed in triplicate for each of the formulations.

### 2.3. Mechanical Properties

A texture analyser, TA.XT Stable Microsystems (Surrey, UK), was used to measure the mechanical properties of the formulations, such as gel strength (compressibility) and hardness (firmness). A 10-mm cylindrical Delrin probe (Stable Microsystems, Surrey, UK) was attached to the texture analyser and used to perform the analysis. The formulation was pre-gelled, placed in a cylindrical beaker (width 2 cm) and maintained at 37 °C using a Peltier cabinet. The probe was set to penetrate the gel with a force of 5 g at a speed of 2 mm/sec for a depth of 5 mm. Exponent 32 software was used to plot a force versus distance graph. This graph was used to calculate the gel strength and hardness. Measurements were performed in triplicate for each of the formulations.

### 2.4. Rheological Analysis

The rheological analysis was performed using an AR-G2 rheometer (TA instruments, Melbourne, Australia) equipped with a temperature-controlled Peltier plate. A stainless parallel plate geometry (40 mm) in oscillatory mode was used to perform all the rheological measurements [29]. The rheological studies were conducted at 37 ± 0.1 °C and 20 ± 0.1 °C. The linear viscoelastic region (LVR) was determined by measuring the storage modulus (G’) and loss modulus (G”) as a function of the strain sweep over the amplitude range of 0.1–100%.

A frequency sweep was then performed to determine the viscoelastic nature of the formulation. G’ and G” were compared for each formulation between an angular frequency range of 1–100 rads^−1^ [30]. The flowability of the gel was determined by carrying measurement of viscosity (η) as a function of shear rates between 0.1 and 200 s^−1^.

### 2.5. In Vitro Gel Degradation Studies

The in vitro gel degradation studies were conducted in phosphate-buffered saline (PBS), and the temperature was maintained at 37 °C using Eppendorf Thermomixer C (Hamburg, Germany). Briefly, a known quantity of formulation was weighed and transferred to an Eppendorf tube. The formulations were allowed to gel at 37 °C. After gelation was achieved, 1 mL of prewarmed PBS (37 °C) was layered over the surface of the gelled formulation. The Eppendorf tubes were placed in a thermomixer maintained at 37 °C. The media was completely removed at predetermined time intervals, and the tube was weighed. This was followed by layering fresh-warmed PBS (1 mL) on the remaining amount of gel and was performed until the entire gel degraded into the media. The study was performed in triplicates for each formulation. The weight difference was used to calculate the gel degradation rate.

### 2.6. In Vitro Drug Release Studies

In vitro drug release studies were conducted in PBS at 37 °C with a shaking speed of 20 rpm and under sink conditions [24]. A known amount of formulation was injected directly into the bottom of a falcon tube and allowed to gel at 37 °C inside the water bath. PBS was prewarmed to 37 °C in the same water bath. The release media (40 mL) was then carefully added over the formulations after they gelled completely. At predetermined time points, 500 μL of sample was withdrawn and was replaced with prewarmed PBS to maintain the sink conditions. The release study was carried out for a period of 3 days. A triplicate analysis was performed for all the formulations and samples were analysed for the LF content using a developed and validated HPLC method.

### 2.7. Osteoblast Viability

Rat osteoblasts were isolated from 20-day foetal rat calvariae, as previously described [31]. Briefly, the frontal and parietal bones of calvariae were collected, free of suture and periosteal tissue. The calvariae were sequentially digested using collagenase, and the cells from digests 3 and 4 were pooled. Cells were grown in T75 flasks in DMEM containing 10% foetal bovine serum (FBS), 5-μg/mL ascorbate-2-phosphate (A2P) and penicillin–streptomycin (P/S). After two days, the media was changed to Minimum Essential Medium (MEM) containing 10% FBS, A2P and P/S and grown to 90% confluency. Cells were then seeded into 24-well plates in MEM with 5% FBS, A2P and P/S for 24 h before treatment began at a density of 2.5 × 10^4^ cells/well.

Poloxamers were found to completely disintegrate in culture media within days. Therefore, to assess cytocompatibility, LF-loaded poloxamers (formulation LF200/poloxamer, 250-µL gel) were placed in tissue culture inserts (1-μm pore size, Greiner Bio-one, Kremsmünster, Austria) and suspended above the well. Poloxamer gel was UV-sterilised after loading in inserts. Viability of the primary rat osteoblasts was assessed following 24 and 72 h using the alamarBlue^®^ assay by adding 5% alamarBlue^®^ (final concentration in well) to each well for four hours at 37 °C before the end of the culture. At the end of the incubation, 200 μL of the alamarBlue^®^ conditioned medium was transferred from each well to a 96-well plate, and the fluorescence was measured. Fluorescence (excitation 540 nm and emission 630 nm) was read using a Synergy 2 multi-detection microplate reader (BioTek Instruments Inc., Winooski, VT, USA). For analysis, the results were normalised to the fluorescence readings from blank wells with media and alamarBlue^®^ only.

### 2.8. Immune Response with THP-1 Cells

The safety of LF/poloxamers was determined by assessing the immune response of THP-1 human monocyte-like cells. Poloxamer gels were UV-sterilised after being placed on cell culture plates. THP-1 cells were maintained and seeded in 10% FBS/RPMI-1640 + 1-mM sodium pyruvate media on top of gelled poloxamers at a density of 1.5 × 10^6^ cells/well. Cells were collected after 24 and 48 h of treatment with LF/poloxamers. RNA was harvested, purified using the TRIzol and Direct-zol™ RNA Miniprep (Zymo Research, Irvine, CA, USA), followed by a gene expression analysis of proinflammatory cytokines by real-time PCR (QuantStudio 12K Flex Real-Time PCR System, Applied Biosystems, Beverly, MA, USA).

Suture material (ETHICON^®^ Coated VICRYLTM Polyglactin 910, Johnson & Johnson International, Raritan, NJ, USA) that is commercially available and clinically used was added into the well without poloxamers to have a guideline for an acceptable immune response. Sterile suture material was cut into 5-cm lengths each and placed into wells of a 24-well plate. The suture was submerged in 70% ethanol for 30 min and then soaked in serum-free RPMI media (Invitrogen, Waltham, MA, USA) for 1 h for pre-equilibrium.

### 2.9. Rat Calvarial Defect Model

All animals were obtained from the Vernon Jansen Unit at the University of Auckland, New Zealand. The surgical procedures were carried out as described previously [6]. Briefly, rats were premedicated with carprofen (10 μL/g) prior to surgery. Once the rats were appropriately anaesthetised with isoflurane, a 5-mm-diameter, bicortical, extra-dural defect was created over the left parietal bone using a trephine burr (Komet Dental, Lemgo, Germany). The periosteal flap was then repositioned over the defects. Following skin closure, 0.2 mL of marcaine (1.25 mg/mL solution) was injected around the surgical site for postoperative analgesia. There was no evidence of local or systemic infection in any of the rats throughout the perioperative period.

Postoperatively, the rats were housed singularly and transferred to a warming cabinet overnight for recovery. Rats were weighed and monitored, and carprofen (10 μL/g) and saline (2 mL) were administered subcutaneously twice daily for 2 days postoperatively for analgesia and fluid replacement. The rats were weighed and monitored until the end of the experiment.

To study bone regeneration, the defects were created in 44 sexually mature male Sprague–Dawley rats weighing over 250 g. The rats were randomised into the following groups for two experimental time points of 4 and 12 weeks postoperation: empty defects (Control, *n* = 10 for each time point), defects grafted with LF200/poloxamers (*n* = 10 for each time point) and sham group (*n* = 2 for each time point, midline incision made through skin and periosteum down to the parietal bone without creation of a bony defect). LF/poloxamer gels (100 μL; containing 20-μg LF) were allowed to set at 37 °C for at least an hour before surgical implantation into the calvarial defects. The defect created over the parietal bone was grafted by pasting the LF/poloxamer gel over the defect site. At the predetermined endpoint, the rats were euthanised with CO_2_ inhalation. The calvaria containing the parietal lobes and parts of the frontal and occipital lobes were excised.

### 2.10. MicroCT

Samples were randomised, scanned and analysed in a blinded fashion. The calvaria were fixed in 10% neutral buffered formalin at 4 °C for three days, then transferred to 70% ethanol. Samples were rehydrated in PBS overnight before the scan. MicroCT scanning (Skyscan 1172, Bruker, Kontich, Belgium) was performed with X-ray voltage 60 kV, 1.0-mm aluminium and copper filters and a voxel size of 12 µm. Scanned files were reconstructed using NRecon software; the datasets were analysed using CTAn software (Bruker). The volume of interest (VOI) was created over the defect site in a cylinder shape with a 5-mm diameter to measure the regenerated bone within the defect area. The beam-hardening effect was corrected for both in the scan settings and during the reconstructions. Tissue mineral density was calculated from measurements of the X-ray attenuation of bone tissue, excluding the pores, and calibrated to hydroxyapatite standards. Two calvarial samples from the 4-week timepoint and three samples from the 12-week timepoint were excluded from analysis due to damage to the calvarial bone during tissue excision before the treatment groups were unblinded.

### 2.11. Histology

Rat calvaria, after microCT, were decalcified in 10% formic acid for 7–12 days and stored in 70% ethanol until paraffin-embedded using a Leica Embedder and a Leica APS 300S automated tissue processor (Leica Biosystems, Richmond, IL, USA). Rat calvarial sections were cut at the mid-point of the defects in the coronal plane. Sections 10 μm thick were cut, and the sections were stained with haematoxylin and eosin (H&E). The whole histological sections were imaged using a MetaSystems VSlide slide scanner (MetaSystems, Altlussheim, Germany). Individual images were stitched into a large image using V-Slide software, Metafer Slide Scanning Platform and Metaclient.

For evaluation of the histological sections of calvariae, a histology grading system was used to generate semiquantitative outcomes for the quantity and quality of regenerated bone within the defect (Table 1). The cross-sections of the calvariae were evaluated by two blinded musculoskeletal scientists, and the average scores were reported. The following histology scoring table was used for the evaluation.

### 2.12. Statistical Analysis

Data were analysed using GraphPad Prism Software version 8.0.2 (GraphPad Software, San Diego, CA, USA). Data were analysed using one-way or two-way analyses of variance (ANOVA) with post hoc Dunnett’s multiple comparison tests when more than two groups were compared or by Student’s *t*-test when two groups were compared. *p*-values of less than 0.05 were considered significant. Data are presented as the mean ± standard error of mean (SEM), standard deviation (SD) or 95% confidence interval (CI). In vitro experiments were repeated at least three times for each assay using different biological samples.

## 3. Results

### 3.1. In Vitro Characterisation of Poloxamer Gels

Poloxamer-based in situ gels containing varying concentrations of LF (Table 2) were successfully developed and characterised for their in vitro properties.

#### 3.1.1. Sol-to-Gel Transition Temperature and Time

The sol-to-gel transition (gelation) temperature and time required for complete gelation of the developed formulations are presented in Table 2. As presented, all formulations demonstrated phase transition in less than a minute in the temperature range of 28.5–29.5 °C. Of note, the increase in LF concentration was associated with a slight rise in gelation temperature and time (*p* < 0.05).

#### 3.1.2. Mechanical Properties

The mechanical properties, such as gel strength and gel hardness, of the developed poloxamer gel formulations are presented in Table 2. An indirect relationship was observed between the LF concentration and gel mechanical properties. Formulation LF10/poloxamer, containing the lowest concentrations of LF (10 µg/mL), demonstrated the highest gel hardness and gel strength. Whereas formulation LF200/poloxamer, containing 200 μg/mL of LF, had the lowest values amongst all the other formulations.

#### 3.1.3. Rheological Properties

**Figure 1:**
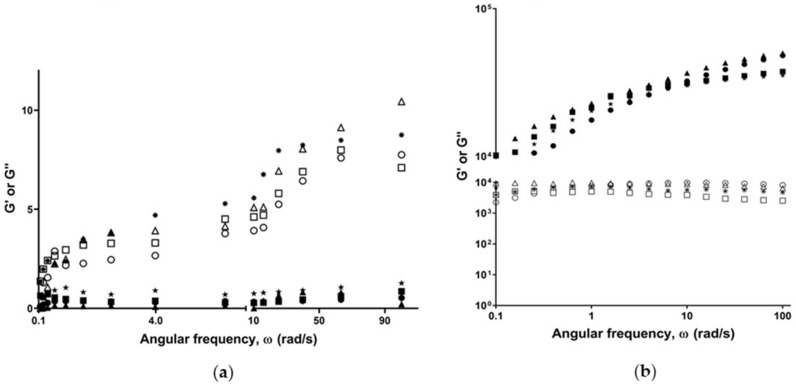
Frequency sweep measuring storage/elastic (G’) and loss/viscous (G”) moduli as a function of angular frequency (ω) at (**a**) 20 °C and (**b**) 37 °C. G’ represented as filled symbols and G” as unfilled symbols. The formulations represented as LF10/poloxamer (*), LF50/poloxamer (■), LF100/poloxamer (●) and LF200/poloxamer (▲).

Study of the rheological properties of poloxamer gels is crucial to ensure their suitability for the intended purpose. All formulations demonstrated linear viscoelasticity at low strain amplitudes (0.1–1%), followed by a sharp reduction of their elastic modulus. Flow behaviour analysis demonstrated that the developed formulations exhibited a Newtonian-like flow at 20 °C and behaved as pseudoplastic materials at 37 °C. Findings of the frequency sweep study (Figure 1) revealed that all formulations exhibited a dominant viscous (G”) moduli at 20 °C, as compared to the elastic moduli (G’). On the contrary, (G’) was significantly higher at 37 °C, as compared to (G”).

#### 3.1.4. In Vitro Gel Degradation Profile

**Figure 2:**
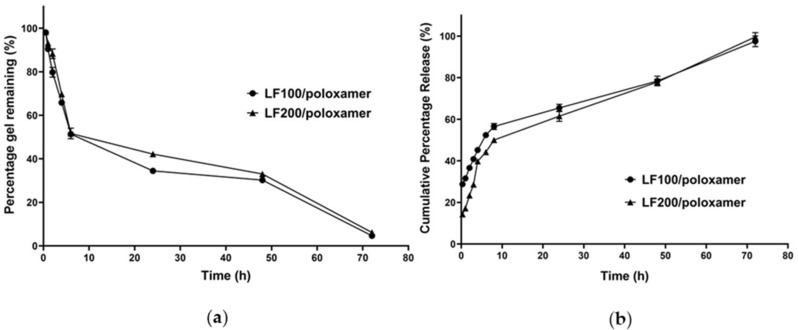
(**a**) Gel degradation and (**b**) cumulative percentage in vitro release profiles of poloxamer gel formulations loaded with LF. Data shown are the mean ± SD (*n* = 3).

Formulation LF100/poloxamer and LF200/poloxamer were chosen for performing in vitro studies, as they had displayed phase transition at a suitable temperature, as well as the LF release characteristics during the first 6 h similar to a previous successful enhanced bone regeneration study [6]. The in vitro gel degradation profile is presented in Figure 2a. It is observed that approximately 50% of the formulations degraded in the first 6 h, and complete degradation of the gel was achieved in 3 days. After the initial burst, the erosion slowed down, with approximately 15% eroding in the next 24 h. The formulation LF200/poloxamer showed slow erosion, with 42.23 ± 1.32% remaining after 24 h compared to the LF100/poloxamer formulation, which had only 34.5 ± 0.972% remaining. However, the overall degradation profiles of the two formulations were very similar without significant differences.

#### 3.1.5. In Vitro Release Profile of LF from Poloxamers

As presented in Figure 2b, the formulations LF100/poloxamer and LF200/poloxamer demonstrated an initial burst release of 56.53 ± 1.46% and 50.02 ± 1.35%, respectively, within the first 8 h, followed by a slower and steady release for up to 72 h. Notably, a statistical analysis revealed that the percentage cumulative LF release from the LF200/poloxamer was significantly lower (*p* = 0.0012) than the LF100/poloxamer within the first 6 h, which could be attributed to its relatively slow erosion rates (Figure 2a).

To identify the LF release mechanism, the Korsmeyer–Peppas mathematical model was applied to the obtained release data. As presented in Table 3, drug diffusion was the dominant mechanism for LF release from both the formulations, as *n* values below 0.45 were achieved [33,34]. Of note, the LF200/poloxamer demonstrated a smaller release constant (K) than the LF100/poloxamer, denoting the slower release rate of LF from the LF200/poloxamer.

### 3.2. Effect of Poloxamers on Osteoblast Viability

**Figure 3:**
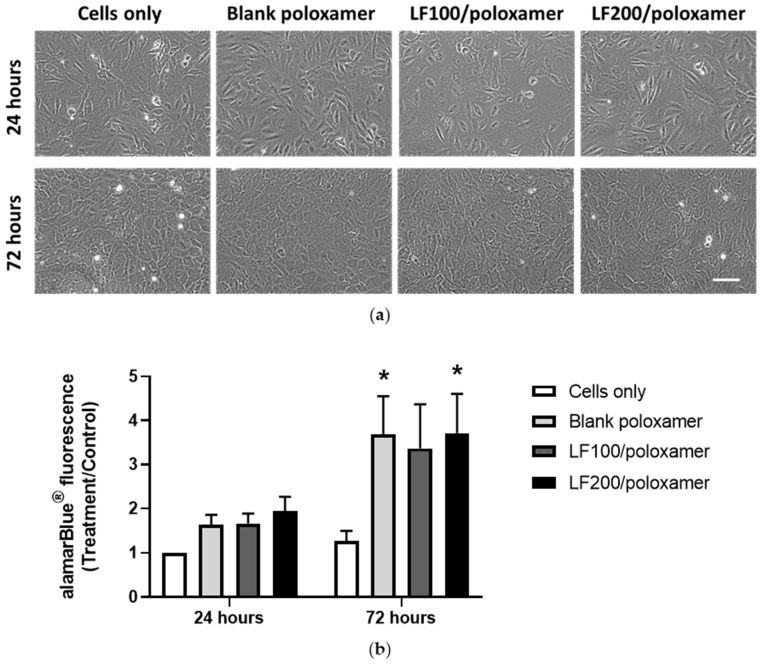
Poloxamers increase primary rat osteoblast viability at 72 h in the presence or absence of LF. (**a**) Representative phase images of osteoblasts. Scale bar = 200 µm. (**b**) Osteoblast viability results at 24 and 72 h. Results from three biological experiments were pooled together (*n* = 3 for each experiment). The cells-only group had inserts hanging on the well without any gel added in the inserts. The 200-µL poloxamers gel was added to each insert. Data shown are the mean ± SEM; two-way ANOVA with post hoc Dunnett’s test, * *p* < 0.05 versus the cells-only group (control) at that time point.

The formulations the LF100/poloxamer and LF200/poloxamer were placed in inserts hanging above the osteoblast culture wells, so that the products released from both gel and cells could be exchanged through the porous membranes of the inserts. The effect of poloxamers on the osteoblast viability was evaluated by examining the cell viability in the absence (blank poloxamer) and presence of poloxamers and with varying LF concentrations (formulation LF100/poloxamer and LF200/poloxamer). The cell viability was determined by examining the cell morphology and measuring the viability of the osteoblasts after 24 and 72 h. As presented in Figure 3a, there was no difference in cell morphology between the groups. Interestingly, poloxamers, regardless of the presence or absence of LF, increased the alamarBlue^®^ readings at 72 h. The presence of LF in the gel had no negative effect on the cell viability (Figure 3b). It was observed that the poloxamers started to break down at 24 h and completely disintegrated at 72 h.

### 3.3. Immune Response to Poloxamers

**Figure 4:**
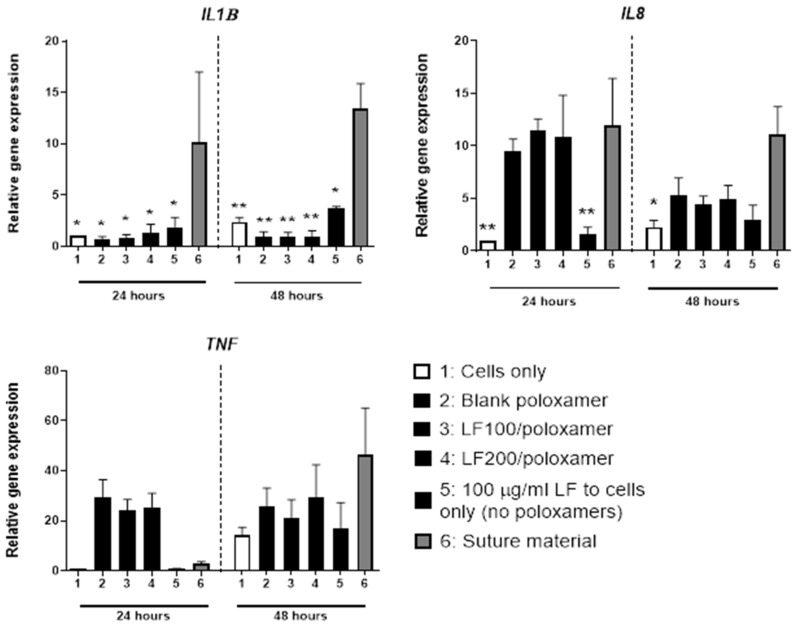
The expression levels of genes encoding inflammatory cytokines in THP-1 cells treated with LF-loaded poloxamer gels. Representative proinflammatory cytokine markers were examined. The cells were cultured on 200-μL poloxamers for 24 and 48 h. Results from three biological experiments were pooled. Data shown are the mean ± SEM; two-way ANOVA with post hoc Dunnett’s test versus suture materials at that time point; * *p* < 0.05 and ** *p* < 0.01.

To investigate if the poloxamers were likely to induce an immune response, changes in the mRNA expression levels of inflammatory markers were evaluated in THP-1 cells cultured with poloxamers. The levels of genes encoding key proinflammatory cytokines IL-1β, IL-8 and TNF-α were measured as indicators of an immune response. As an estimate of acceptable immune response, the gene expression levels of the inflammatory markers were compared to the level of surgical suture materials used clinically. The mRNA expression of *IL1B, IL8* and *TNF* for the groups with poloxamers were similar or lower (*p* < 0.05) than the levels expressed by cells cultured with suture materials at 48 h (Figure 4).

### 3.4. In Vivo Results

#### 3.4.1. MicroCT

**Figure 5:**
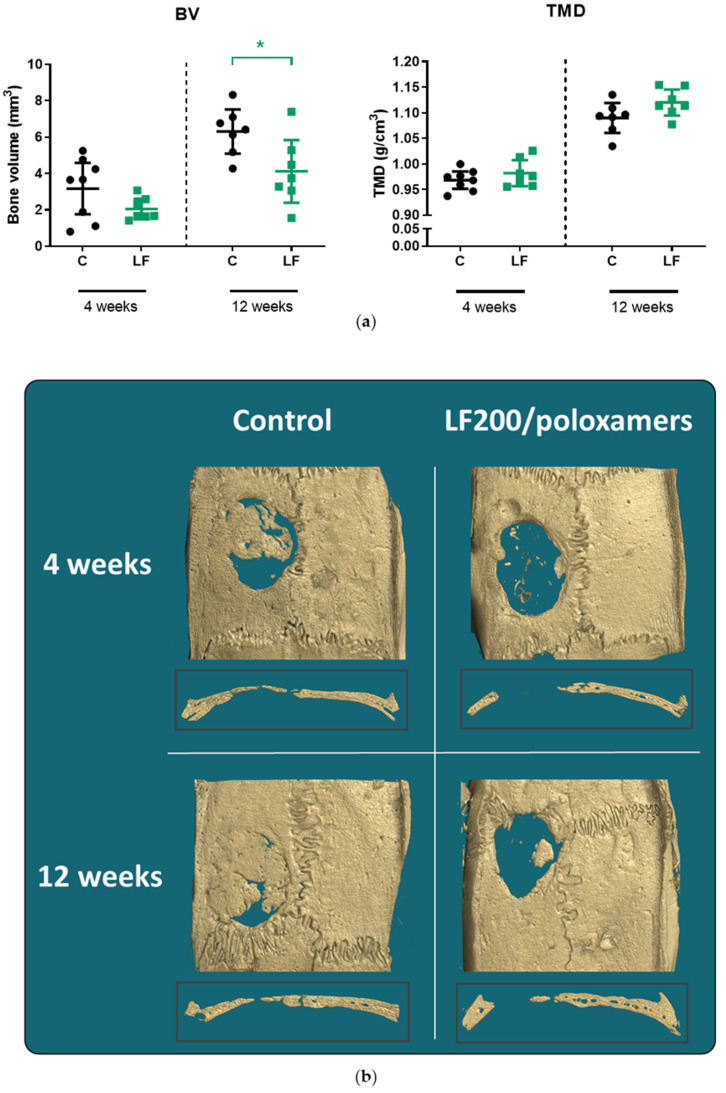
MicroCT results of rat calvariae at 4 and 12 weeks postoperation. (**a**) Bone volume and tissue mineral density of the control and LF200/poloxamer groups. C, Control; LF, LF200/poloxamer; BV, bone volume; TMD, tissue mineral density. Data shown are the mean ± 95% CI; two-way ANOVA with post hoc Dunnett’s test, * *p* < 0.05 versus the control at that time point. (**b**) Reconstructed bone images of rat calvariae using the microCT data. The median bones from the bone volume data in each group are presented with cross-section at the middle of the defect area.

At four weeks after surgery, there was no difference in the mean bone volume (BV) and tissue mineral density (TMD) between the control and LF200/poloxamer groups (Figure 5). At 12 weeks, the group treated with the LF200/poloxamer had lower mean BV (*p* = 0.0435) than the control group, but the TMD was similar in the two groups (Figure 5).

#### 3.4.2. Histology

**Figure 6:**
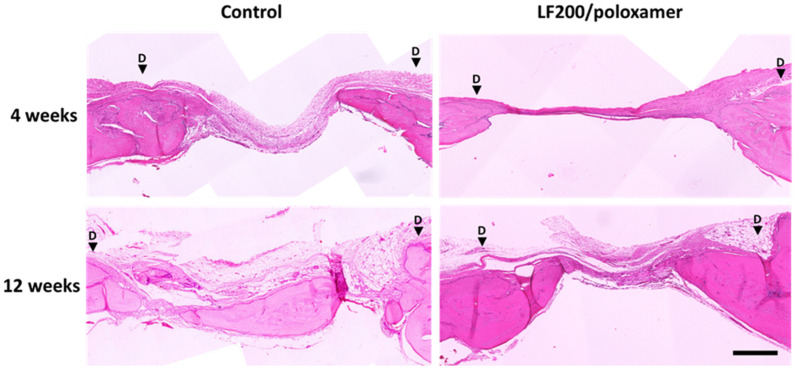
Histology evaluation of regenerated bone at 4 and 12 weeks postoperation. Representative histology section of regenerated bone. The calvariae sections were stained with H&E. The calvariae that were bisected coronally so the cross-section of the defect in the middle can be seen. The black arrows indicate the boundaries of the defects. Scale bar = 500 µm.

At 12 weeks, bones from the control group had a higher defect coverage score (*p* = 0.0267) compared to bones from the LF200/poloxamer group. A higher score indicated a higher percentage of the defect area was covered by new woven bone, appearing at the edge of the detect site, in control bones compared to the LF200/poloxamer-treated bones, consistent with the microCT results (Figure 6). There was no difference in the ‘New bone type’ or ‘Neo-vascularisation’ scores between the groups at both the 4- and 12-week time points (Table 4). There was no increase in the inflammation scores in the LF200/poloxamer groups at either time point, as suggested by the gene expression (mRNA levels were below the level of the suture material group) (Figure 4).

## 4. Discussion

Our study found that poloxamer formulations could be loaded with lactoferrin that was released gradually over three days in an in vitro setting. The poloxamer formulations support cell viability in vitro and are not likely to induce an unfavourable immune response. However, in vivo, the current formulation of LF200/poloxamers failed to support bone regeneration in the rat calvarial defect model.

For an in-situ gelling system to be suitable for administration, it should maintain its liquid status at room temperature to allow ease of administration and transit to the gel status at physiological temperature. As presented in Table 2, all formulations exhibited a sol-to-gel transition in a suitable temperature range >25 °C and <37 °C, suggesting their suitability for the intended purpose. Interestingly, the inclusion of LF increased the sol-to-gel transition temperature and time, which could be attributed to its physical/chemical interactions with poloxamers. Noteworthy, formulations with shorter sol-to-gel transition time are likely to exhibit a shorter lag time between administration and gel formation, with a potential to reduce the initial burst release [35].

In agreement with the literature, poloxamers demonstrated the linear viscoelastic region (LVR) at a low-strain amplitude. The findings suggest that the developed formulations can maintain their internal structural integrity within 0.1–1% strain amplitude. Therefore, the viscoelastic properties were studied at a strain amplitude of 0.5%. As presented in Figure 1, all formulations exhibited significantly higher viscous moduli at 20 °C, confirming the liquid status at the test conditions. On the other hand, they exhibited higher elastic moduli at 37 °C, denoting the transition to the gel status.

In vitro release studies are crucial to understand/predict the in vivo performance of the developed formulations. As presented in Figure 2b, an initial burst release of LF was observed from the LF100/poloxamer and LF200/poloxamer formulations during the first 6 h, followed by a sustained release for up to 72 h. In the cell culture plate, the gel was observed to be disintegrated at the end of the study. Combined with the kinetic modelling result in this study (Figure 1 and Figure 2 and Table 3), this suggests that the release of LF is controlled by gel erosion during the 72 h following an initial burst release of LF by diffusion [24].

Many studies investigated the two components in our poloxamers formulation: P407 and P188. The poloxamers were reported to be biocompatible with different cell types and had low toxicity and immunogenicity [14]. We carried out in vitro experiments to confirm that the combination of poloxamers used in the gel preparations were biocompatible with bone cells, and it was observed that the bone cells viability was maintained in the presence of poloxamer gel formulations (LF100/poloxamer and LF200/poloxamer). The levels of the key proinflammatory cytokines were also measured to test if the poloxamers would induce any inflammation in vivo. The natural healing process involves an increased expression of the cytokines, including IL-1 and TNFα, which are involved in the initiation of a cascade of repair events [36]. The expression of those cytokines peaks within the first 24 h of injury during the natural healing process, which is also shown in our results, with an upregulation of *TNF* mRNA levels in the presence of the poloxamers within the first 24 h. This upregulation was followed by a decrease in the expression levels similar to the level of surgical suture material by 48 h, which would be considered acceptable. Based on the gene expression results in Figure 4, both the LF100/poloxamer and LF200/poloxamer were well-tolerated by THP-1 cells and did not induce an increase in the inflammatory cytokine gene expression in vitro. In addition, when compared to the surgical suture material, the response was mild, and therefore, the poloxamers were tested in vivo.

The in vivo results suggested that, at 4 weeks, there was no difference in bone regeneration between the two groups. At 12 weeks, the LF200/poloxamers attenuated bone regeneration compared to the control group. When bone regeneration within each group was compared between the two time points, both groups had doubled the amount of BV and increased the TMD from 4 to 12 weeks. The histology scores also confirmed the microCT results that the quality of regenerated bone in the two groups were similar when the three categories (new bone type, vascularisation and the presence of inflammation) were taken into consideration. This may suggest that the poloxamers slowed bone regeneration. It is less likely that the poloxamers had a toxic effect on the osteoblasts at the defect site, as suggested by our in vitro results and as the poloxamer P407 has been shown to support human osteoblast-like cell MG-63 viability and differentiation [37] and osteoblasts in vivo [38,39].

One interesting study that used poloxamer P407 as a carrier for the demineralised bone matrix (DBM) examined the effect of DBM delivered in the poloxamer or water on mesenchymal cell differentiation and measured ALP-stained cells up to 21 days [40]. The group treated with DBM in the poloxamer had lower ALP activity compared to the group treated with DBM in water, suggesting the poloxamer had a negative effect on the differentiation of the mesenchymal cells. The authors speculated that the presence of a poloxamer between the DBM particles negatively affected the release of growth factors from the DBM, inhibiting the mesenchymal cell differentiation. In our study, it is possible that the presence of poloxamers at the defect site at the beginning of the healing stage negatively affected the initial healing process, as the initial stage of bone healing involves the presence of extensive cell signalling and growth factors. The results from the DBM study suggest that a poloxamer may push the cells away from differentiation and towards proliferation, as our results showed increased osteoblast viability in the presence of poloxamers. However, in our study, we did not evaluate the differentiation of osteoblasts when cultured with our formulation of poloxamers. Therefore, it is possible that the reduced bone regeneration seen in vivo was a result of the inhibition of osteoblast differentiation by poloxamers.

A limitation of our in vitro study was that we were not able to assess if the poloxamers are compatible when the cells are physically in contact with the gel. The standard osteoblast proliferation assay runs for at least 72 h, and by that time, the gel is already degraded in the cell culture media, so it was not possible to place the cells on top of the gels. To overcome this problem, we measured the osteoblast viability using inserts suspended over the cell culture wells (as described in the Methods). This method provides information regarding the degradation products of the poloxamers, which may be of more relevance. Another limitation of our in vivo study was the absence of a group treated with a poloxamer alone. The study showed that critical-sized calvarial defects in rabbits filled with poloxamer P407 were identical to the defects left unfilled when assessed clinically and radiographically [41], suggesting the non-osteoinductive property of poloxamers. Despite that, the poloxamer-only group could have added to our study, especially with our results showing a negative effect of the LF200/poloxamer in bone regeneration.

It is worth noting that the calvaria is not a weight-bearing bone, and the calvarial defect model is a widely used system with some advantages for studying the effect of growth factors or delivery systems. It is a good system to study the effect of factors without the effect of weight-bearing, which is known to have an effect on bone remodelling. The poloxamers have not been examined in a long bone defect model yet, but it is worth noting that the two types of bones involve different mechanisms of fracture healing, including endochondral and intramembranous bone healing and different fracture repair time, and hence, the effect of LF in different bone models could show different results.

It should also be considered that, in the present study, the poloxamer formulations were gelled in advance, and then, the gels were pasted over the bone defect site, which was very different from the target administration methods for this in situ gel delivery system. In situ gel systems are meant to be injected using a syringe and the needle at the defect site. This change in the administration method of the poloxamer formulation might have influenced the in vivo results. The change might have also influenced the gel strength and hardness, which play a crucial role in defining the release profile and predicting the in vivo performance of poloxamer gels [42,43]. Further studies involving Fourier-transform infrared spectroscopy or Raman spectroscopy would identify the type and extent of interactions between LF and poloxamers in the formulations gelled in a different method.

Although the developed poloxamer gel formulation LF200/poloxamer demonstrated promising results in vitro, with increased cell viability and less likely to induce immune responses, the formulation failed to enhance bone regeneration in the in vivo study. With previous studies showing the positive effect of LF on local bone regeneration, we speculate this formulation is not compatible with the rat calvarial defect model, and therefore, a different animal model of bone regeneration should be explored in the future, along with further formulation optimisation to enhance local bone regeneration.

## Figures and Tables

**Table 1:** Histology grading system used to semi-quantify outcomes of regenerated bone within defects modified from Musson et al. [32].

Score	Bone Defect Coverage % of Defect Filled (Volume) by New Bone within Defect Area	New Bone Type Nature of New Bone within Defect	Neo-Vascularisation Presence of Vascularisation within the Newly Formed Bone	Inflammation Presence of Inflammatory Cells around the Newly Formed Bone
0	0%	No new bone	No evidence of neovascularisation	Abundant inflammation and evidence of encapsulation
1	1–24%	Predominantly woven	Few new vessels (<10)	Relatively few or normal amount of inflammatory cells present
2	25–49%	1:1 mix of woven and lamella	Abundant neovascularisation	-
3	50–74%	Predominantly lamella remodelled	-	-
4	75–100%	-	-	-

**Table 2:** Sol-to-gel transition temperature, time and mechanical properties of developed poloxamer gel formulation loaded with LF. Poloxamer composition: P407 25% *w*/*w* and P188 17% *w*/*w*. Data presented as the mean ± SD (*n* = 3).

Formulation Code	LF Conc. (μg/mL)	Gelation Temperature (°C)	Gelation Time (sec)	Gel Strength (g·s)	Gel Hardness (g)
LF10/poloxamer	10	28.5 ± 0.5	35	74.95 ± 6.87	34.46 ± 2.00
LF50/poloxamer	50	29.0 ± 0.5	45	52.61 ± 3.54	26.96 ± 1.72
LF100/poloxamer	100	29.0 ± 0.0	50	45.37 ± 2.39	21.10 ± 3.89
LF200/poloxamer	200	29.5 ± 0.5	55	34.41 ± 0.99	16.36 ± 0.50

**Table 3:** Release kinetic parameters of LF from the poloxamer gel formulations.

Formulation Code	r^2^	*n*	K
LF100/poloxamer	0.9732	0.2305	87.9631
LF200/poloxamer	0.9716	0.2604	72.4465

**Table 4:** Histology evaluation of regenerated bone. Histology grading of regenerated bone within defect area in the control and LF200/poloxamer groups based on the criteria set out in Table 1. Two independent researchers blinded to the treatment groups scored the histological sections. Data shown are the mean ± SEM; Student’s *t*-test. * *p* < 0.05 compared to the control. *n* = 10 in each group.

	Bone Defect Coverage		New Bone Type		Neo-Vascularisation		Inflammation	
	4 Weeks	12 Weeks	4 Weeks	12 Weeks	4 Weeks	12 Weeks	4 Weeks	12 Weeks
Control	2.2 ± 0.40	3.15 ± 0.38	1.45 ± 0.20	2 ± 0.17	1.8 ± 0.11	1.65 ± 0.13	0.55 ± 0.16	0.45 ± 0.12
LF200/poloxamer	1.7 ± 0.28	1.95 * ± 0.32	1.25 ± 0.15	1.7 ± 0.20	1.7 ± 0.11	1.35 ± 0.15	0.65 ± 0.13	0.3 ± 0.11

## Data Availability

The data presented in this study are available on request from the corresponding author.
